# Supplementary material for: Modulating electrospun polycaprolactone scaffold morphology and composition to alter endothelial cell proliferation and angiogenic gene response
Source: PLoS One. 2020 Oct 8;15(10):e0240332. doi: 10.1371/journal.pone.0240332 (PMC7544109; doi:10.1371/journal.pone.0240332)
Supplement: Attachment — Submitted filename: Reviewer Comments.docx [file pone.0240332.s003.docx]

Reviewer Comments

1. The author states in the introduction that ‘fibre diameter on endothelial cells has not yet been studied’. However, there has been ample research done on the effects of fiber diameter on endothelial cell behavior.

Some of the references are

- Rüder, Constantin, Tilman Sauter, Karl Kratz, Tobias Haase, Jan Peter, Friedrich Jung, Andreas Lendlein, and Dietlind Zohlnhöfer. "Influence of fibre diameter and orientation of electrospun copolyetheresterurethanes on smooth muscle and endothelial cell behaviour." Clinical hemorheology and microcirculation 55, no. 4 (2013): 513-522.
- Ko, Young-Gwang, Ju Hee Park, Jae Baek Lee, Hwan Hee Oh, Won Ho Park, Donghwan Cho, and Oh Hyeong Kwon. "Growth behavior of endothelial cells according to electrospun poly (D, L-lactic-co-glycolic acid) fiber diameter as a tissue engineering scaffold." Tissue engineering and regenerative medicine 13, no. 4 (2016): 343-351.

The authors need to cite appropriate references and remove claims that are not justified.

2. The authors need to check the paper for typographical errors and grammatical mistakes. Eg. Superscript -1 in the sentence ‘Coherent anti-stokes Raman scattering (CARS) imaging at 2911cm-1 was used to image the PCL scaffold fibres, whilst simultaneously exciting two photon fluorescence (TPEF) from Phalloidin and DAPI stained cells’.
3. The authors need to clarify on how the diameters of fibers were measured. How many FESEM images for each sample were used to obtain the statistical significance with regard to fiber diameter? The difference between the 3um and 4um fibers are quite close. Is it possible that the diameters might have not been statistically significant if other images of the same sample were used?
4. The authors need to elaborate on the reproducibility of the electrospinning results. Environmental factors play a major role in the diameter and the subsequent fiber morphology.
5. The authors have performed a one-way ANOVA. Why did the authors choose ANOVA and not regression-based analysis?
6. The mechanical properties and stiffness of the fibers also changes along with diameter. Why have the authors chosen to just represent that fiber diameter is the sole factor leading to changes in expression? The authors need to think about performing AFM if possible to get a better understanding about the scaffold properties.
7. The thickness of the fabricated scaffolds needs to be mentioned in the main paper. Differences in cell infiltration can only be compared when the thickness of the scaffolds are similar. The variable thickness could also be a factor in expression of genes. The author needs to further investigate and elaborate on the different variables.
8. There are no appropriate controls for the cell titer blue viability assays. A positive and negative control needs to be represented. A standard tissue culture is an ideal positive control.
9. Controls need to be established for the gene expression studies.
10. Surface characterization of the fibers need to be performed in order to demonstrate that the surface properties are similar across all scaffold morphologies.
